# Disease Manifestation and Viral Sequences in a Bonobo More Than 30 Years after Papillomavirus Infection

**DOI:** 10.3390/pathogens8010013

**Published:** 2019-01-26

**Authors:** Markus Hoffmann, Enrika Schütze, Andreas Bernhard, Lennart Schlaphoff, Artur Kaul, Sandra Schöniger, Stefan Pöhlmann

**Affiliations:** 1 Infection Biology Unit, German Primate Center—Leibniz Institute for Primate Research, 37077 Göttingen, Germany; mhoffmann@dpz.eu (M.H.); lennart.schlaphoff@stud.uni-goettingen.de (L.S.); akaul@dpz.eu (A.K.); 2 Institute of Veterinary Pathology, University of Leipzig, 04103 Leipzig, Germany; enrika.schuetze@vetmed.uni-leipzig.de (E.S.); sandra.schoeniger@vetmed.uni-leipzig.de (S.S.); 3 Zoo Leipzig GmbH, 04105 Leipzig, Germany; abernhard@zoo-leipzig.de; 4 Faculty of Biology and Psychology, University of Göttingen, 37073 Göttingen, Germany

**Keywords:** pygmy chimpanzee, papillomavirus, sequence evolution, focal epithelial hyperplasia

## Abstract

Pan paniscus Papillomavirus 1 (PpPV1) causes focal epithelial hyperplasia (FEH) in infected animals. Here, we analyzed the present disease manifestation and PpPV1 genomic sequence of an animal that was afflicted by an FEH epizootic outbreak in 1987 for which the sequence of the responsible PpPV1 was determined. The animal displayed FEH more than 30 years after the initial diagnosis, indicating persistence or recurrence of the disease, and evidence for active PpPV1 infection was obtained. Moreover, the sequences of the viral genomes present in the late 1980s and in 2018 differed at 23 nucleotide positions, resulting in 11 amino acid exchanges within coding regions. These findings suggest that PpPV1-induced FEH might not undergo complete and/or permanent remission in a subset of afflicted animals.

## 1. Introduction

Papillomaviruses contain a double-stranded circular DNA genome of approximately 8 kb that is surrounded by a shell of viral proteins. The family *Papillomaviridae* harbors about 130 species, divided into the subfamilies *Firstpapillomavirinae* (52 genera) and *Secondpapillomavirinae* (one genus) [1]. The viruses are mainly transmitted via the sexual route or direct skin-to-skin-contact and infect basal cells in the squamous epithelium of mucosal surfaces and skin [2]. Infection of skin and anogenital mucosa with low risk human papillomaviruses (HPV), including HPV-6 and HPV-11, may result in the development of benign lesions. In contrast, infection with high risk HPV, including HPV-16 and HPV-18, is associated with a significantly elevated risk for malignant lesions, including cervix carcinoma [2].

Apart from anogenital mucosa, HPV can also infect the oral mucosa and infection might be associated with potentially malignant disorders, including oral leukoplakia, oral erythroplakia, oral proliferative verrucous leukoplakia, oral submucous fibrosis, oral lichen planus, and actinic cheilitis [3]. Moreover, oral HPV infection can cause benign lesions, including squamous cell papilloma, condyloma acuminatum, verruca vulgaris, and focal epithelial hyperplasia (FEH, also termed Heck’s disease) [3]. FEH has mainly been observed among children and adolescents of American Indian origin [4,5] and in Inuit [6,7], but has occasionally also been detected in other populations. Males and females are equally affected by FEH and genetic predisposition seems to impact susceptibility to disease [3,8,9]. FEH lesions are round or ovoid, can heal spontaneously and are most frequently observed in the labial and buccal mucosa [3,10]. Finally, HPV-13 and HPV-32 infections were found to be associated with FEH [11,12].

An outbreak of FEH in a colony of bonobos (pygmy chimpanzees, *Pan paniscus*) was observed in a zoological garden in 1987 and was found to be associated with an HPV13-related virus, Pan paniscus Papillomavirus 1 (PpPV1, https://pave.niaid.nih.gov/; previously termed pygmy chimpanzee papillomavirus 1) [13,14]. PpPV1 and HPV-13 share 85% similarity at the nucleotide level and display an identical genome organization [13]. Several aspects of PpPV1 infection and PpPV1-induced FEH are poorly understood. Thus, when FEH went into partial (two out of five animals) or complete remission (three out of five animals) in the afflicted bonobos after the first detection of lesions in 1987, it remained unknown whether the animals with partial remission ultimately became disease free [13,14]. Moreover, PpPV1 sequence diversity has not been investigated. We obtained a sample from a bonobo with FEH (as determined by gross examination) that was submitted for virus diagnostics and analysis of the donors records revealed that it was part of the bonobo colony in which PpPV1 had initially been identified in the late 1980s [14]. This afforded the opportunity to investigate whether viral infection was still active and whether disease manifestation and viral genome sequence had changed over a period of almost 30 years.

Here, we report that the animal suffered from FEH, suggesting that either the disease did not undergo full remission or recurred, and that viral antigens were detectable in the oral mucosa, demonstrating active viral infection. Moreover, we show that the viral genome sequence had a total of 23 nucleotide exchanges, 11 of which resulted in amino acid changes compared to the reference genome that was obtained from the initial outbreak in 1987 [14].

## 2. Materials and Methods

### 2.1. Sample Acquisition

On 30 November 2017, tissue samples from a lesion in the oral cavity of a bonobo (*Pan paniscus*) that was kept at Leipzig zoological garden, Germany, was sent to the German Primate Center for clarification of the cause for the tissue alterations. Upon arrival, the tissue sample was halved. One part was placed in 10% buffered formalin for a microscopic examination and the other part was used for PCR (polymerase chain reaction) examination. 

Samples from a bonobo housed in a zoological garden for public display were analyzed in the present study. Institutional policies of the zoological garden require that animal health is monitored daily. Monitoring revealed that the bonobo had difficulties with ingestion. In order to determine the underlying reasons, the oral cavity was examined. Samples were taken from the anesthetized animal by certified veterinarians and then subjected to diagnostic tests for viral infections. Approval by an ethics committee or institutional review board was not required.

### 2.2. Histological and Immunohistochemical Examinations

Formalin-fixed biopsy samples of the oral lesion were dehydrated, embedded in paraffin wax, cut into 4 µm sections, and processed for histopathology and immunohistochemistry. The histopathological examination was performed on haemalaun and eosin (H&E) stained sections. For immunohistochemistry, a mouse monoclonal antibody crossreactive against various HPV, including HPV-13, was employed as primary antibody (LifeSpan BioSciences, Seattle, WA, USA). The immunostaining was performed with the peroxidase-antiperoxidase (PAP) method and 3.3´-diaminobenzidine tetrahydrochloride (DAB) as a chromogen [15]. In detail, tissue sections were dewaxed, rehydrated, and incubated with 3% H_2_O_2_ in methanol for 30 minutes (min) at room temperature (RT) to block the endogenous peroxidase activity. The primary antibody (1:150) was incubated overnight at 4 °C. This was followed by several washing steps and the application of rat anti-mouse immunoglobulin as a bridge antibody (1:100, Dianova, Hamburg, Germany, 30 min, RT). Sections were thoroughly rinsed, treated with mouse-PAP-complex (1:500, Dianova) for 30 min at RT, washed again, and incubated in DAB (10 min). Sections were counterstained with Papanicolaou’s solution. 

In the negative control, the primary antibody was replaced with a non-binding isotype-matched control antibody.

The antibodies and the PAP-complex were diluted with tris-buffered saline (TBS) containing 1% bovine serum albumin (BSA). For all washing steps, TBS was used.

### 2.3. PCR-Based Detection of PpPV1

Total DNA of unfixed tissue (≈50 mg tissue) was extracted by means of a First-DNA All-Tissue Kit (GEN-IAL GmbH, Troisdorf, Germany) as recommended by the manufacturer (elution volume, 100 µL). Next, the extracted DNA was quantified using a NanoDrop ND-1000 spectrophotometer (Peqlab, Erlangen, Germany) and further subjected to a PCR protocol that detects a broad range of papillomaviruses. The PCR-mix was prepared on ice and consisted of the following components: 100 ng extracted DNA, 1.2 µM forward primer (FAP64, 5′-CCWATATCWVHCATITCICCATC-3′) [16], 1.2 µM reverse primer (FAP6085, 5′-CCWGATCCHAATMRRTTTGC-3′) [17], 200 µM dNTPs (each), 2 mM magnesium chloride, 5% DMSO (dimethyl sulfoxide), 2.5 µL 10× reaction buffer, and 1.25 U HotStar Taq DNA Polymerase (QIAGEN, Hilden, Germany). DEPC (diethyl pyrocarbonate)-treated water was used for equilibration to a total volume of 25 µL and the PCR was subsequently performed under the following conditions (a reaction that contained water instead of DNA was used as a negative control): one cycle at 95 °C for 4 min, 30 sec; 55 cycles consisting of 95 °C for 20 sec, 46 °C for 30 sec, and 72 °C for 30 sec; and one cycle at 72 °C for 10 min. Reactions were then mixed with 5 µL of 6× Purple Loading Dye (New England Biolabs, Ipswich, MA, USA), and subjected to agarose gel electrophoresis using a 1.5% agarose gel containing 1 µg/mL ethidium bromide. After that, the gel was imaged under UV light and a band of the expected size (≈380 bp) present in the analyzed sample was excised from the gel, purified using the NucleoSpin Gel and PCR Clean-up kit according to manufacturer’s protocol (Macherey-Nagel, Düren, Germany) and send to a commercial sequencing service (Microsynth SeqLab, Göttingen, Germany) for Sanger sequencing using the primers used for amplification. The resulting sequences were assessed for quality by inspecting the chromatograms. Further, the resulting sequences were analyzed using the basic local alignment search tool for nucleotide sequences (BLASTn; National Center for Biotechnology Information, Bethesda, MD, USA) and revealed 100% sequence identity to X62844.1 (Pygmy Chimpanzee papilloma virus type 1 DNA, now termed Pan paniscus Papillomavirus 1) after the removal of primer-specific sequences.

### 2.4. Amplification and Sequencing of DNA Fragments Covering the Whole PpPV1 Genome

After confirmation that the sample was positive for PpPV1, we designed primers for PCR amplification of overlapping fragments of the published genome found under the GenBank entry X62844.1 (Table 1). All reactions were prepared on ice and consisted of the following components: 100 ng extracted DNA, 1 µM forward primer, 1 µM reverse primer, 200 µM dNTPs (each), 5% DMSO, 5 µL 5× HF buffer and 1 U Phusion High-Fidelity Polymerase (Thermo Fisher Scientific, Waltham, MA, USA). DEPC-treated water was used for equilibration to a total volume of 25 µL and the PCR was subsequently performed under the following conditions: one cycle at 96 °C for 2 min; 35 cycles consisting of 96 °C for 30 sec, 54 °C for 30 sec, and 72 °C for 90 sec; and one cycle at 72 °C for 10 min. Reactions were then mixed with 5 µL of 6× Purple Loading Dye (New England Biolabs), and subjected to agarose gel electrophoresis using a 1.0% agarose gel containing 1 µg/mL ethidium bromide. After that, the gel was imaged under UV light, and the obtained bands were excised from the gel, purified, and sent for sequencing. 

To minimize the risk of sequence errors resulting from false nucleotide incorporations by the polymerase during PCR, we utilized a polymerase with proof-reading activity and each nucleotide of the PpPV1 genome was covered by at least two individual PCRs (fragments are summarized in Table 2). Further, we verified every single nucleotide of the PpPV1 genome by sequencing reactions based on at least two individual amplicons and sequenced both the + and the − strand.

The full PpPV1 genome was assembled from the individual sequencing reads and aligned to the published reference genome (X62844.1) using MEGA6 (https://www.megasoftware.net/) [18]. The obtained PpPV1 genome has been submitted to GenBank and can be accessed via the accession number MK303593.

## 3. Results

**Figure 1:**
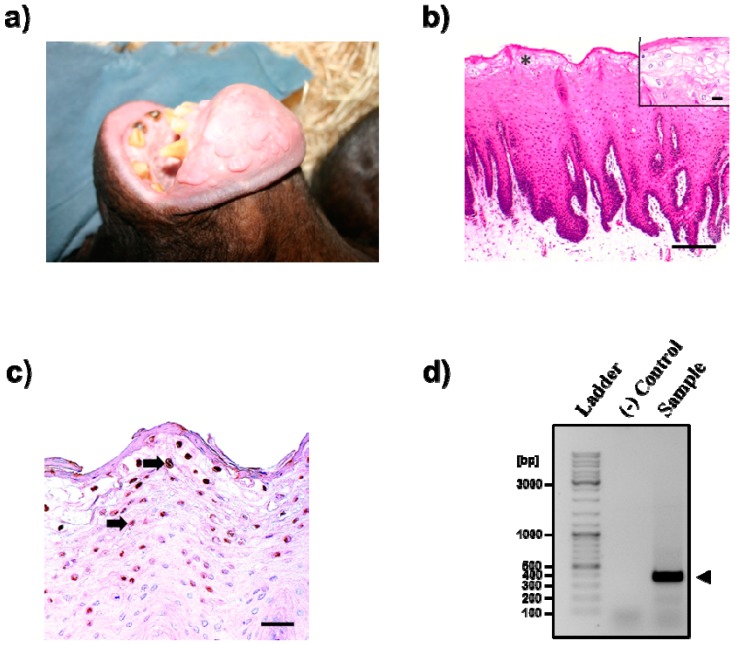
Focal epithelial hyperplasia and papillomavirus infection in the oral cavity. (**a**) Macroscopic findings of focal epithelial hyperplasia in the oral cavity. (**b**) Histological features of focal epithelial hyperplasia: There is marked irregular epithelial hyperplasia with formation of distinct rete ridges. The pallor of the superficial layers (asterisk) is caused by the presence of numerous koilocytes. Bar, 200 µm. Inset: Koilocytes that are characterized by cytoplasmic vacuolation are depicted at a higher magnification. Bar, 20 µm. Haemalaun & eosin stain. (**c**) Immunostaining for papillomavirus antigen: several keratinocytes of the stratum spinosum including koilocytes contained immunopositive nuclei (arrows). Bar, 50 µm. (**d**) PCR-based papillomavirus detection in the tissue sample from the oral cavity of the bonobo. Lane 1, DNA ladder (GeneRuler DNA Ladder Mix, Thermo Fisher Scientific); lane 2, negative control (water was used instead of DNA for PCR); lane 3, DNA from tissue sample. The arrowhead indicates the positive band at ≈380 bp. Small numbers on the left side indicate the size of selected reference bands of the DNA ladder (given as base pairs, bp).

The animal, a 36-year-old male bonobo born in a zoological garden in Antwerp [13,14], was immobilized due to difficulties with ingestion. Visual inspection of the oral cavity revealed two broken incisors as well as lesions. The lesions had a cauliflower-like and nodular shape and were detected on the interior lip, vestibules, and gingivae but not on tongue and soft and hard palate (Figure 1a). Histopathological examination revealed multifocal areas of marked irregular epithelial hyperplasia (acanthosis) with distinct rete ridges and the presence of numerous koilocytes in superficial epithelial cell layers. The latter represent keratinocytes with a prominent cytoplasmic vacuolation (Figure 1b). Rarely, degeneration of individual epithelial cells was observed. The stratum corneum showed mild to moderate parakeratosis. A mild infiltration with lymphocytes, plasma cells, and neutrophils was present in the epithelium and underlying fibrovascular connective tissue. Numerous keratinocytes, including koilocytes of the stratum spinosum, showed a positive nuclear immunostaining for the PV antigen (Figure 1c). Finally, PCR with FAP64 and FAP6085 primers specific for a region in the L1 gene of papillomaviruses [16,17] amplified a ≈380 bp fragment expected for HPV genome (Figure 1d), and Sanger sequencing in combination with sequence analysis confirmed that the sequence was identical to that previously published for PpPV1 (not shown). In sum, our results confirmed a PpPV1 infection and show that FEH did only partially regress after its first diagnosis in 1987.

**Figure 2:**
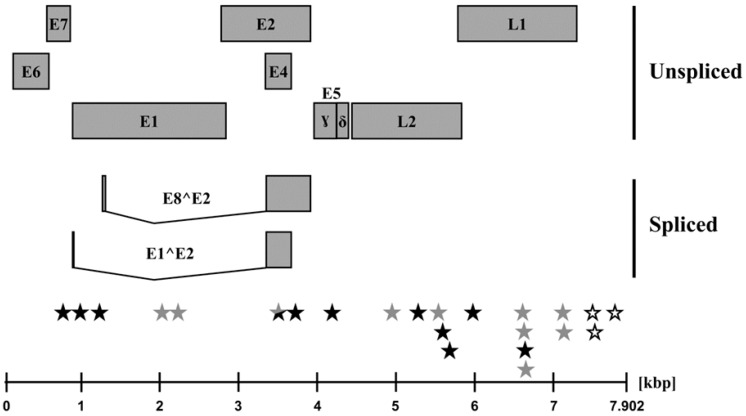
Schematic representation of the Pan paniscus Papillomavirus 1 (PpPV1) genome and location of the identified nucleotide variations. PpPV1 contains a circular, double-stranded DNA genome of 7902 bp that codes for a total of eleven proteins from different transcriptional frames (for simplicity, the circular genome is shown in a linearized fashion). Nucleotide differences in comparison to the original sequence (X62844.1) are highlighted between the schematic genome and the scale. Black stars indicate nucleotide differences that led to amino acid changes in open reading frames (ORFs) of the genome, while grey and white stars refer to nucleotide differences that do not translate into amino acid changes and that were located in non-translated regions, respectively. kbp, kilobase pairs.

At present, sequence evolution and diversity of PpPV1 are largely unknown. Therefore, we sought to compare the viral genomic sequences present at the time of first diagnosis in the late 1980s and detected within the present study. For this, we PCR-amplified a total of 18 overlapping fragments spanning the entire viral genome and determined the sequences of the complete + and − strand. Sequence analysis showed that genomic sequences identified in the present study and those deposited in GenBank in 1991 (X62844.1, the original sequence has been updated in 2005) differed in 23 nucleotides, resulting in a sequence identity of 99.71% (Figure 2 and Table 3). Three mutations were located in the non-translated region located at the 3’ end of the L1 open reading frame. In contrast, 20 changes were found in coding regions, and of those 11 resulted in a change in the amino acid sequence (Table 3). The highest number of amino acid changes was observed in the L2 protein (n = 3), while two changes were detected in both the E1 and L1 protein. Finally, single amino acid changes were detected in the E5ɣ and E7 proteins (Table 3).

## 4. Discussion

Infection of the oral mucosa by HPV and its consequences are incompletely understood, although detection of high risk HPV in buccal mucosa has been reported and infection by HPV-13 and HPV-32 has been shown to be associated with FEH [11,12,19,20]. Here, we report continued FEH in a bonobo that has been first diagnosed with the diseases in 1987 and reported the genomic sequence of the causative agent, PpPV1.

The animal investigated in the present study, a 36-year-old bonobo named Joey, was first diagnosed with FEH in 1987 [14]. At this time, Joey was part of colony of eight bonobos that were born in different places (Kinshasa, Stuttgart, and Antwerp) and that were co-housed in a zoological garden in Antwerp. Five out of the eight animals were diagnosed with FEH in 1987, while three were disease free [14]. Within the next approximately three to four years, three out of the five animals with FEH became disease free [14], in keeping with the observation that FEH due to HPV-13 infection can undergo complete remission in human patients [3,10]. In contrast, Joey and another animal showed incomplete remission approximately four years after FEH diagnosis [14]. In keeping with the incomplete remission of FEH in Joey, we observed no lesions on tongue and hard palate, although these areas contained lesions in 1987. However, it is unknown whether lesions remained constant over decades or recurred after periods of complete remission. Finally, it should be stated that the bonobo colony in Antwerp has not been subject to follow-up studies, although FEH was reported in an animal imported from Antwerp to San Diego [21], and that Joey is currently co-housed with 11 bonobos, of which the FEH status is unknown. 

The detection of viral proteins in lesions suggested persistent infection and the PpPV1 genome could be readily PCR amplified from buccal mucosa samples. This allowed us to determine its sequence. The PpPV1 sequence obtained differed in 23 nucleotides from the sequence reported in 1992 [13] and 11 of the 23 changes resulted in amino acid substitutions in viral proteins. The lack of adequate cell lines and functional assays precluded testing whether these changes altered virus–host cell interactions. Moreover, the reasons for the notable sequence differences could not be elucidated. However, we can offer several speculations. First, the differences in the present PpPV1 sequence and the sequence reported in 1992 might reflect inter-host diversity, which can be substantial for papillomaviruses [22,23]. Thus, it has not been documented whether the PpPV1 sequence reported in 1992 was obtained from material from Joey or another infected bonobo from the same colony and it cannot be excluded that the FEH outbreak observed in the bonobo colony in 1987 was due to two or more parallel introductions of PpPV1 from different sources. Moreover, it is conceivable that Joey was infected with a different PpPV1 variant after the FEH was epizootic in the colony in Antwerp, since co-infection with different papillomavirus types can occur despite mechanisms preventing superinfection being operative in infected cells [24]. Second, the sequence differences might be due to intra-host evolution. This scenario seems unlikely, since the papillomavirus evolutionary rate is believed to be too low to allow analysis via viral passaging in cell culture or via studying sequential samples taken from an infected host [25,26,27]. However, we note that recent studies suggest substantial intra-host diversity of HPV at given time points after infection [28,29]. Moreover, consecutive samples from Joey are not available but would be required to demonstrate sequence evolution. Third, it cannot be excluded that a fraction of the nucleotide changes observed might be due to PCR errors, as sequencing technology has improved since the time when the initial study was performed. With respect to this, we would like to point out that in the present study, both the + and the – strand were sequenced and an enzyme with proof-reading activity was used for PCR amplification of PpPV1 sequences. 

## 5. Conclusions

Our study indicates that FEH induced by PpPV1 might not undergo remission in some animals and provides insights into PpPV1 sequence diversity or evolution.

## Figures and Tables

**Table 1:** Primers designed for amplification of fragments covering the Pan paniscus Papillomavirus 1 (PpPV1) genome.

Name	Sequence (5′–3′)
PpPV1_1F	CAGCTCAGAAGAAGATGAGG
PpPV1_1R	CTTAGTAATACTAACAATAGC
PpPV1_2F	TGCATCATAGTGTGTCTGAGG
PpPV1_2R	TTCTGTCAAAAGGGAATGG
PpPV1_3F	TAGAAAACATAAATCGTTAGC
PpPV1_3R	CCTGTGCAAGGGTGGTTGTGG
PpPV1_4F	AGCAAAGTTATATGTTCTCC
PpPV1_4R	CTGCGAGGCCTACTATGTGC
PpPV1_5F	TTACTGCATATTAGAACTGG
PpPV1_5R	CGTCCACCAATATGTTTGCC
PpPV1_6F	GGCTATAACGTCTAGGCGTGG
PpPV1_6R	ACACCTGAACATTGTGTGCC
PpPV1_7F	GTTTGGTGTGGGCCTGTATAGG
PpPV1_7R	AAACTGAGACCTGCATAAGG
PpPV1_8F	TTTAGAAGAATGGAACTTTGG
PpPV1_8R	GAAACCGTTTTCGGTCCCTCC
PpPV1_9F	AATCCTTTTTGGCTACCAGC
PpPV1_9R	ATGTCCGATCCTGTACAGTCC

**Table 2:** Amplicons produced to cover the Pan paniscus Papillomavirus 1 (PpPV1) genome.

Primer For	Primer Rev	Amplicon	Amplicon Size (bp)	Genome Region (nt)
PpPV1_1F	PpPV1_1R	1a	1055	618–1672
	PpPV1_2R	1b	2005	618–2622
PpPV1_2F	PpPV1_2R	2a	1062	1561–2622
	PpPV1_3R	2b	1825	1561–3385
PpPV1_3F	PpPV1_3R	3a	909	2477–3385
	PpPV1_4R	3b	1914	2477–4390
PpPV1_4F	PpPV1_4R	4a	1102	3289–4390
	PpPV1_5R	4b	2038	3289–5326
PpPV1_5F	PpPV1_5R	5a	1046	4281–5326
	PpPV1_6R	5b	1989	4281–6269
PpPV1_6F	PpPV1_6R	6a	1036	5234–6269
	PpPV1_7R	6b	1843	5234–7076
PpPV1_7F	PpPV1_7R	7a	1051	6026–7076
	PpPV1_8R	7b	1927	6026–50
PpPV1_8F	PpPV1_8R	8a	1020	6933–50
	PpPV1_9R	8b	1728	6933–758
PpPV1_9F	PpPV1_9R	9a	912	7749–758
	PpPV1_1R	9b	1826	7749–1672

**Table 3:** Summary of the identified nucleotide variations and their effect on protein level.

Genome		Genetic Element	Open Reading Frame			
Position (nt)	Effect (nt) ^a^		Position (nt)	Position (aa)	Effect (aa) ^b^	Codon ^c^
711	G711C	E7	180	60	Q60H	CAx
861	A861G	E1	28	10	K10E	xAG
1170	A1170C	E1	337	113	I113L	xTA
1964	T1964C	E1	1131	377	silent (F)	TTx
2172	A2172C	E1	1339	447	silent (R)	xGG
3472 ^d^	C3472G	E2	751	251	R251G	xGA
3666 ^e^	C3666A	E2	945	315	D315E	GAx
3472 ^d^	C3472G	E4	189	63	silent (L)	CTx
3472 ^d^	C3472G	E1^E4	165	55	silent (L)	CTx
3666 ^e^	C3472G	E8^E2	187	63	R187G	xGA
3472 ^d^	C3666A	E8^E2	381	127	D381E	GAx
4136	G4136C	E5ɣ	237	79	L79F	TTx
4883	C4883A	L2	516	172	silent (P)	CCx
5217	G5217A	L2	850	284	V284I	xTA
5477	G5477A	L2	1110	370	silent (S)	TCx
5523	A5523C	L2	1156	386	K386Q	xAG
5613	G5613A	L2	1246	416	C416Y	TxT
5900	G5900A	L1	155	52	R52K	AxA
6543	G6543A	L1	798	266	silent (E)	GAx
6549	A6549T	L1	804	268	silent (I)	ATx
6555	A6555C	L1	810	270	E270D	GAx
6573	G6573T	L1	828	276	silent (G)	GGx
7071	C7071T	L1	1326	442	silent (L)	CTx
7089	T7089C	L1	1344	448	silent (N)	AAx
7443	G7443C	URR	n.a.	n.a.	n.a.	n.a.
7469	A7469C	URR	n.a.	n.a.	n.a.	n.a.
7726	C7726G	URR	n.a.	n.a.	n.a.	n.a.

a: Nucleotide difference compared to the reference sequence (X62844.1); b: Effect of the nucleotide variation on protein level (for silent mutations, the letters written in brackets indicate the respective amino acid residue); c: The affected nucleotide is indicated by the letter “x”; d: Identical genome position (the coding region for E2, E4, E1^E4, and E8^E2 partially overlap); e: Identical genome position (the coding region for E2 and E8^E2 partially overlap); Abbreviations: nt = nucleotide, aa = amino acid residue, URR = upstream regulatory region, n.a. = not applicable.
